# Relating adaptive genetic traits to climate for Sandberg bluegrass from the intermountain western United States

**DOI:** 10.1111/eva.12240

**Published:** 2015-01-28

**Authors:** Richard C Johnson, Matthew E Horning, Erin K Espeland, Ken Vance-Borland

**Affiliations:** 1 Plant Germplasm Research and Testing, United States Department of Agriculture, Agricultural Research ServicePullman, WA, USA; 2 Deschutes National Forest, United States Department of Agriculture Forest ServiceBend, OR, USA; 3 Pest Management Research Unit, United States Department of Agriculture, Agricultural Research ServiceSidney, MT, USA; 4 Conservation Planning InstituteCorvallis, OR, USA

**Keywords:** climate, genecology, plant adaptation, *Poa secunda*, restoration, seed zones

## Abstract

Genetic variation for potentially adaptive traits of the key restoration species Sandberg bluegrass (*Poa secunda* J. Presl) was assessed over the intermountain western United States in relation to source population climate. Common gardens were established at two intermountain west sites with progeny from two maternal parents from each of 130 wild populations. Data were collected over 2 years at each site on fifteen plant traits associated with production, phenology, and morphology. Analyses of variance revealed strong population differences for all plant traits (*P *<* *0.0001), indicating genetic variation. Both the canonical correlation and linear correlation established associations between source populations and climate variability. Populations from warmer, more arid climates had generally lower dry weight, earlier phenology, and smaller, narrower leaves than those from cooler, moister climates. The first three canonical variates were regressed with climate variables resulting in significant models (*P *<* *0.0001) used to map 12 seed zones. Of the 700 981 km^2^ mapped, four seed zones represented 92% of the area in typically semi-arid and arid regions. The association of genetic variation with source climates in the intermountain west suggested climate driven natural selection and evolution. We recommend seed transfer zones and population movement guidelines to enhance adaptation and diversity for large-scale restoration projects.

## Introduction

In the U.S., exotic species and native cultivars have been extensively used over large geographic areas for restoration. Over the last 20 years, there has been an increasing emphasis on the use of local native plant germplasm to maintain local adaptation and ecological relationships when restoring landscapes (Erickson 2008). Although the use of native, local plant materials is increasingly desired, an understanding and implementation of seed zones—geographic boundaries for appropriate germplasm movement—is lacking for most native plant species.

As climate varies across the landscape, it is reasonable to expect that heritable genetic traits in growth and development will evolve (Turesson 1922; Clausen et al. 1940; Aspinwall et al. 2013). Genecology is a way to develop seed zones by analysing how genetic variation of populations within a species relate to their source environments (Johnson et al. 2012, 2013; St. Clair et al. 2005, 2013). Genecological studies use common garden experiments to quantify genetically based phenotypic differences among a relatively large number of plant populations (Campbell 1986; Rehfeldt 1994; St. Clair et al. 2005, Johnson et al. 2010, 2012, 2013) and to assess environmental factors as a potential selective force driving local adaptation (Kawecki and Ebert 2004; Miller et al. 2011, Weißhuhn et al. 2012). Plants from different source climates exhibiting differences in adaptive growth and development traits suggests adaptation though natural selection (Endler 1986; Kawecki and Ebert 2004).

Although exceptions have accumulated (Galloway and Fenster 2000; Leimu and Fischer 2008; Bischoff and Müller-Schärer 2010; Hancock et al. 2012), locally derived germplasm has often been shown to have an adaptive advantage (Hufford and Mazer 2003; Rice and Knapp 2008), and in the absence of supporting data, use of local germplasm is a reasonable precautionary approach against restoration failure. In a survey of 74 reciprocal transplant studies, Hereford (2009) found the overall frequency of local adaptation measured as relative fitness was 0.71, and differences between home site environments were positively associated with the magnitude of local adaptation. In a similar survey, Leimu and Fischer (2008) found the same result, but also found that local adaptation was much more common in larger than smaller populations. Compared to genecology based on common garden studies, reciprocal transplant studies more directly evaluate adaptation in terms of differences in fitness and performance, but unlike common gardens, only relatively small number of experimental test sites and seed sources can normally be evaluated over a region of interest (Galloway and Fenster 2000; Kawecki and Ebert 2004). As a result, landscape scale reciprocal transplant studies as conducted by Wang et al. (2010) on lodgepole pine (*Pinus contorta* Dougl. ex Loud.) are rare. Common gardens are way to assess genetic variation among populations with a relatively large number of seed sources (Kawecki and Ebert 2004).

In restoration, the common practice of seeding selected ecotypes over large and varied geographic areas has the potential to promote genetic swamping, genetic erosion (Hufford and Mazer 2003; Johnson et al. 2010), outbreeding depression (Kramer and Havens 2009), and unfavorable interactions with other plant and animal species. Moreover, restoration seedings with exotic species such as the crested wheatgrass (*Agropyron cristatum* L. Gaertn.) have lead to monocultures that can competitively exclude desirable native species (Gunnell et al. 2010) leading to the need to enhance species diversity (Fansler and Mangold 2011). As stressful environments expand with climate change, intraspecies genetic diversity across the landscape is critical to promote *in situ* selection and continued evolutionary potential (Sgrò et al. 2011).

Thus, restoration practices should consider adaption, ecological relationships, and the maintenance of genetic diversity (Rice and Emery 2003; Sgrò et al. 2011). Using a common garden approach and source population climate, genecology is a way to determine the environmental scale of potentially adaptive traits (Galloway and Fenster 2000) and may be the best current approach to guide restoration that promotes genetic diversity and positive ecological relationships over large geographic areas.

The intermountain western United States extends from the Sierra Nevada and Cascade Mountain ranges in the west to the Rocky Mountains in the east. The western mountains block moisture from Pacific storms leaving typically arid to semi-arid landscapes in valleys and plateaus of the intermountain region. Climatic variation in this region leads to desert sage-shrub lands, temperate grasslands, and at higher elevations, montane coniferous forests.

Among the widespread perennial bunchgrass species in the intermountain west is *Poa secunda* J. Presl (sandberg bluegrass), a perennial, facultative apomictic bunchgrass (Kellogg 1987; Kelley et al. 2009). It is a critical component of the sagebrush communities dominant in much of the intermountain west and is commonly planted as a restoration species. Sandberg bluegrass develops relatively early in the spring, is grazed by livestock and wildlife, and the seeds are consumed by birds and small mammals (Majerus et al. 2009). A cycle of increasingly frequent fires and the spread of invasive weeds, especially *Bromus tectorum* L., aggravated by changing climate, is eroding the capacity of these communities to function (Davies et al. 2012; Reisner et al. 2013). In many areas, weed control along with strong restoration programs for sage, bunchgrasses, and herbs are needed to prevent dominance by invasive weeds (Davies et al. 2012). Sandberg bluegrass has shown a level of competiveness, resilience, and adaptation potential to *B. tectorum* invasions (Goergen et al. 2011; Davies et al. 2012), underscoring its role as a key restoration species.

In this study, wild land collections of Sandberg bluegrass from diverse climates in the intermountain west were evaluated in common gardens. Using a genecology approach, our objectives were to (i) assess genetic variation in morphological, phenological, and production traits, (ii) visualize genetic variation as a function of source population climate though regression modeling and GIS mapping, and (iii) develop seed transfer zones to enhance establishment and sustainability of Sandberg bluegrass within restoration plantings over large geographic areas.

## Material and methods

### Population sampling and garden establishment

The Sandberg bluegrass complex has been the subject of taxonomic ambiguity and revision (Majerus et al. 2009). But Kellogg (1985) concluded that although there are local types within the complex, only *Poa curtifolia* (Scibner), a serpentine endemic from central Washington state, represented a separate evolutionary lineage. In this study, germplasm collections corresponded to *Poa sandbergii* from Cronquist et al. (1977) as given by Majerus et al. (2009). This excludes the taller and later summer flowering variants Big bluegrass, Canby's bluegrass, Pacific bluegrass, Nevada bluegrass, and Alkali bluegrass (Majerus et al. 2009).

**Figure 1:**
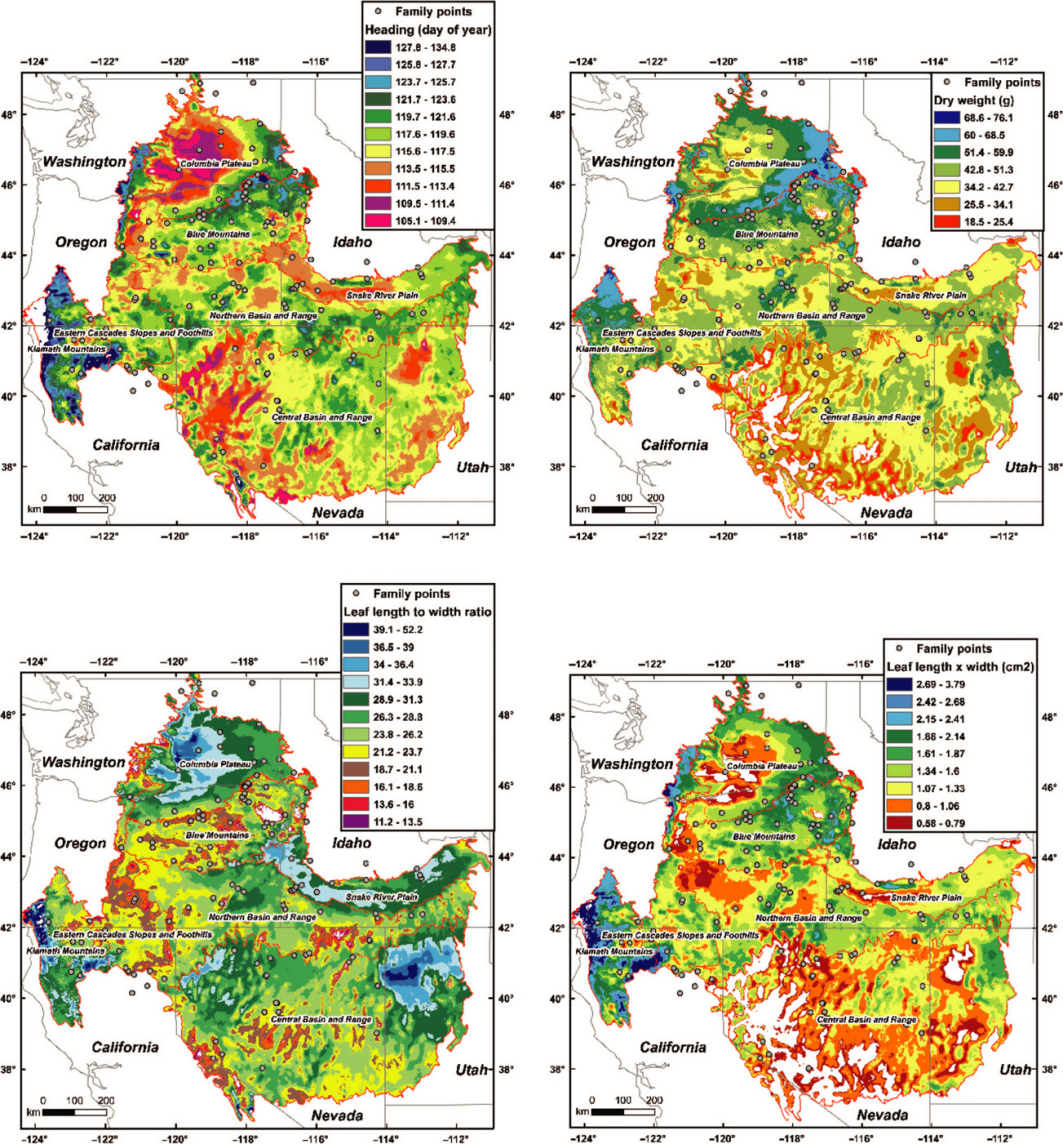
Geographic variation of Sandberg bluegrass plant traits with climate over intermountain west level III Omernik ecoregions and the Klamath Mountain ecoregion. Ecoregion boundaries are shown as red lines, and the circles are the collection source populations. The different colored areas were delimited with contours based on the ± *P* = 0.01 confidence interval from the regression model error. Model predictions outside the trait value data range were not mapped (shown in white).

Seeds from wild plants were collected at 130 source populations from the intermountain west in the spring of 2007 (Fig.1). Seeds from individual plants were collected at each source and maintained separately as families. Latitude, longitude, and elevation were recorded at each source population using geographic positioning instrumentation. For each source population, ‘annual’ climate norms were extracted from ClimateWNA climate data rasters (Wang et al. 2012; http://www.genetics.forestry.ubc.ca/cfcg/ClimateWNA/ClimateWNA.html) for the time period spanning 1981–2010. There were 21 climate variables designated by Wang et al. (2012) as ‘annual variables’ including directly calculated means for temperature and precipitation, warmest and coldest months, summer precipitation, and heat to moisture indices. Additional derived variables included degree day indices, frost free days, precipitation as snow, 30-year minimum and maximum temperature extremes, and evaporative demand indices.

The 130 wild populations together with nine cultivar accessions were established at two common garden sites, the Central Ferry Research Farm, WA (hereafter CF) and the Oregon State University's Central Oregon Agricultural Research Center (COARC) in Powell Butte, OR (hereafter PB). The cultivars were supplied by Benson Farms Inc. (Moses Lake, WA, USA) and Western Reclamation Inc. (Eltopia, WA, USA). Central Ferry is a low elevation (209 m), relatively warm site in the Snake River Canyon and PB a cooler, higher elevation (941 m) site in the high desert of central Oregon. The 30-year (1981–2010) mean annual temperature at CF was 12.0 and at PB it was 9.2°C. The 30-year norm for precipitation at CF is 352 mm and for Powell Butte 269 mm.

In winter, 2008 seeds from the 130 wild accessions and the 9 cultivars were germinated in boxes (13.3 cm long, 12.7 cm wide, and 3.5 cm deep) containing water-saturated vermiculate. The boxes were placed at room temperature (∽20°C) and seeds allowed to germinate. Germinates were planted into 5 × 5 × 5 cm containers in flats holding 36 containers of Sunshine #5 plug mix (SunGro Horticulture, Bellevue, WA, USA) and grown under greenhouse conditions for 6 weeks. Seedlings were watered and fertilized as needed to promote optimal growth. The plants were placed in a lath house for 2 weeks before transplanting.

Seedlings were transplanted at CF on April 8 and at PB on April 22, 2008. Plants were spaced 0.6 m apart in rows separated by 1.5 m at CF; at PB, all plants were spaced 0.6 m apart in all directions. At PB, soil moisture conditions were dry so supplemental irrigation (≈10 mm) was applied the day after transplanting. No fertilizer or additional irrigation was applied at either site.

The 139 germplasm sources were randomized in six complete blocks at both common garden sites. Within each block, wild collections from each source were represented by two families. Thus, there were 6 individuals for each family for a total of 12 from each source population at each site. For cultivars, two plants from each source were included also giving 12 individuals from each source. At each garden site, there were 1668 plants. In 2009 and 2010, after the establishment year, each plant was evaluated for phenology, production, and morphology traits at each site as defined in Table1. This required data collection twice per week at each site during the heading through seed maturity period.

**Table 1:** Summary of analyses of variance for Sandberg bluegrass plant traits in common gardens at Central Ferry, WA and Powell Butte, OR in 2009 and 2010 and representing seed collections from source populations across the intermountain west and surrounding areas

		Site		Population		Site × population	
Trait	Mean	*F*-value	*P*-value	*F*-value	*P*-value	*F*-value	*P*-value
Phenology*	2009						
Heading, day of year	123	923	<0.0001	6.06	<0.0001	2.21	<0.0001
Blooming, day of year	138	902	<0.0001	7.43	<0.0001	3.39	<0.0001
Maturity, day of year	165	201	<0.0001	3.96	<0.0001	2.28	<0.0001
Heading to bloom, days	15.4	3.98	0.070	3.01	<0.0001	3.64	<0.0001
Heading to maturity, days	41.9	43.5	<0.0001	4.57	<0.0001	1.97	<0.0001
Bloom to maturity, days	26.4	35.2	<0.0001	3.57	<0.0001	2.56	<0.0001
Production†							
Survival, %	0.74	0.11	0.750	3.40	<0.0001	1.71	<0.0001
Panicles per plant	73.2	9.36	0.012	3.11	<0.0001	3.17	<0.0001
Dry weight, g	26.6	5.61	0.039	5.24	<0.0001	2.26	<0.0001
Crown area*, cm^2^	54.7	1.63	0.231	3.93	<0.0001	1.59	<0.0001
Morphology‡							
Leaf length to width ratio	25.4	8.06	0.017	3.25	<0.0001	1.45	0.001
Leaf length × width, cm^2^	1.82	9.32	0.012	11.4	<0.0001	1.57	<0.0001
Plant habit, 1 (prostrate) to 9 (upright)	6.09	19.2	0.001	2.46	<0.0001	2.54	<0.0001
Culm length, cm	38.4	45.2	<0.0001	3.29	<0.0001	2.23	<0.0001
Panicle length, cm	10.8	9.59	0.011	4.99	<0.0001	2.01	<0.0001
Phenology	2010						
Heading, day of year	111	313	<0.0001	8.56	<0.0001	2.68	<0.0001
Blooming, day of year	131	1749	<0.0001	8.72	<0.0001	2.04	<0.0001
Maturity, day of year	171	0.340	0.572	4.43	<0.0001	2.64	<0.0001
Heading to bloom, days	19.7	230	<0.0001	4.06	<0.0001	3.26	<0.0001
Heading to maturity, days	59.4	95.7	<0.0001	3.90	<0.0001	2.87	<0.0001
Blooming to maturity, days	39.8	375	<0.0001	4.16	<0.0001	3.54	<0.0001
Production							
Survival, %	0.70	0.220	0.650	3.14	<0.0001	1.66	<0.0001
Panicles per plant	127	12.2	0.005	2.25	<0.0001	2.83	<0.0001
Dry weight, g	110	0.950	0.354	2.48	<0.0001	2.47	<0.0001
Crown area, cm^2^	58.6	184	<0.0001	4.27	<0.0001	2.01	<0.0001
Morphology							
Leaf length to width ratio	28.7	3.99	0.073	2.77	<0.0001	1.12	0.168
Leaf length × width, cm^2^	1.40	1.17	0.304	6.78	<0.0001	1.60	<0.0001
Plant habit, 1 (prostrate) to 9 (upright)	5.54	65.5	<0.0001	2.53	<0.0001	2.58	<0.0001
Culm length, cm	32.4	7.90	0.018	3.18	<0.0001	3.28	<0.0001
Panicle length, cm	10.3	10.6	0.008	5.58	<0.0001	1.40	0.002

* Heading was at complete emergence of a lead panicle from its sheath, blooming was the initial appearance of anthers, and maturity when seed appeared mature in more than half the panicles.

† Panicles on each plant were counted after heading, harvest for dry weight was after maturity to within 4 cm of the soil, and after harvest, crown area was estimated as the product of two perpendicular crown diameter measurements.

‡ Leaf length and width were measured on the last fully emerged leaf of a lead culm after heading, culm length was measured from the plant base to the base of the panicle on a lead culm after full stem extension, and panicle length was measured on a lead culm after full stem extension.

### Statistical analysis

Analysis of variance was completed on each plant trait in Table1 using the mixed procedure (Proc Mixed) in sas/stat version 9.2 as described in Littell et al. (1996). Replicate blocks were nested within sites (PB or CF) and families within source populations. Thus blocks within site and families within populations were assumed to be random. Preliminary analyses included years, confirming the expected significant year effects and complex year interactions with site, population, and site × population. Since year, effects were confounded with the growth of perennial plants over years, and year interactions were complex, we chose to present separate analyses for each year. For each trait evaluated, the pooled families within population variance provided an error term to evaluate the difference among populations. This provided an assessment of genetic variation over a large number of diverse environments (St. Clair et al. 2013). Differences between garden sites, source populations, and their interaction were evaluated within each year and for each trait.

Separate analyses of variance were also completed to compare cultivars and wild populations as groups. Families or plants within source populations were averaged and classified as either cultivars or wild populations. Differences between sites, the classification (cultivars and wild population), and their interaction were tested using block within site as a random effect.

For wild populations, variance components for source population, family within population, and residual error was computed using PROC MIXED for each site and year combination. The percentage of total phenotypic variance attributed to each factor was calculated.

We used canonical correlation (PROC CANCORR in sas/stat version 9.2) to assess the relationship between plant traits measured in common gardens and source population climate variables similar to St. Clair et al. (2005). This resulted in canonical variates or linear combinations of traits and climate that maximized their correlation. Each set of variates for traits and climate represent independent dimensions in data, with the first variate having the highest correlation and the last the lowest (Manly 1986).

Because there were 15 plant traits measured at each site and year, there was a potential to use 60 traits in the canonical correlation analysis, 15 for each of the 4 year-site combinations. Such a high number of potential traits complicate the identification of a small set of canonical variates that explain a high fraction of the variation suitable for modeling with climate variables. Consequently, to simplify the modeling, the number of traits used in canonical correlation was reduced using Pearson correlation analysis of traits for each year–site combination to determine the magnitude and direction of interactions. If the correlations coefficient was highly significant and positive (*P *<* *0.01), indicating reasonable correspondence between traits in both magnitude and direction, data where averaged over environments.

Only significant canonical variates (*P *<* *0.01) as composite plant traits were used for regression modeling and mapping trait variation with seed source climate. Additionally, representative production, phenology, and morphology traits that correlated with canonical correlation scores were also regressed on climate variables and models mapped. Regression modeling between plant traits and climate variables was completed using sas proc reg sas/stat version 9.2. The objective was to find models with the highest predictive value with the fewest number of model parameters (Draper and Smith 1998). Within PROC REG, the *R*^2^ option was used along with the Akaike information criterion (AIC) (Akaike 1969) to minimize over parameterization. For a given trait, all climate variables from each source were initially included in the modeling process. The final model selected was the combination of climate variables that produced the highest *R*^2^ with the lowest AIC statistic. These models maximize prediction capacity with the coefficients and variables functioning as a set. Owing to correlations among variables in the regression, they do not necessarily identify the single most important, independent climate variables for a given trait (Manly 1986).

### Regression model mapping and seed zone development

Spatial mapping of plant traits and canonical variates predicted from regression models was completed using the grid algebra function (raster calculator) of the arcgis 9.3 Spatial Analyst extension (ESRI, Redlands, CA, USA). Raster layers comprised of the relevant climate variables were converted to trait values by multiplying each climatic variable by each associated regression coefficient in the model and summing the results. A mapping contour interval corresponding to the 99% confidence level at regression model means was calculated using the regression model error term. To avoid extrapolation beyond the data, mapping was confined to the range of the observed canonical scores for each trait value.

Seed zones that delineate areas of similar plant traits were created by classifying the first canonical variate into high, medium, and low categories, and if significant (*P *<* *0.01), the second and third variates into high and low categories over the range of the scores. The resulting rasters were then overlaid (St. Clair et al. 2005, 2013; Johnson et al. 2012, 2013). The spatial combination (overlays) for the classifications results in seed zones combining attributes represented by the three canonical variates.

## Results

### anova and variance components of wild populations

There was strong evidence for extensive genetic variation among Sandberg bluegrass across the intermountain West. Analyses of variance of wild collections showed significant source population main effects for all 15 plant traits both years (Table1). Differences between garden sites (*P *<* *0.01) were also observed in seven of the 15 traits in 2009 and nine of 15 traits in 2010. For all traits except the leaf length to width ratio, the garden site × source population interaction was also significant (*P *<* *0.01). Thus, populations differed for plant traits depending on site and source, indicating a degree of plasticity (Table1). Plasticity between years and interactions with years also appeared to be substantial for most traits (data not shown).

The fraction of total variation associated with source populations ranged from 70.2% for blooming at PB in 2009 to 1.8% for crown area at PB in 2010 (Table2). Variation within families ranged from 20.8% of the total variation for heading to blooming at PB in 2009 to essentially zero at PB in 2010. In 2009, at PB family variance slightly exceeded the population variance for culm length and in 2010 for blooming to maturity. Still, population variance was on average more than three times that of the family variance, and as fraction of the population and family variance total, populations accounted for an average of 77% of the variance (Table2).

**Table 2:** Percentage of total variance from source populations and from families within populations for plant traits measured on Sandberg bluegrass at two common garden sites for 2 years

	Central Ferry				Powell Butte						
	2009		2010		2009		2010		Means		
Trait	Pop.	Family	Pop.	Family	Pop.	Family	Pop.	Family	Pop.	Family	Pop./(Pop. + Fam) %
Phenology											
Heading	42.0	18.4	60.8	14.7	58.8	14.4	47.1	2.6	52.2	12.5	80.6
Blooming	47.7	18.5	60.0	7.6	70.2	9.7	36.2	8.2	53.5	11.0	82.9
Maturity	24.5	9.6	18.4	0.4	38.9	7.1	27.7	2.9	27.4	5.0	84.6
Heading to bloom	39.0	20.9	25.8	7.4	29.3	20.8	31.6	0.0	31.4	12.3	71.9
Heading to maturity	27.5	5.5	35.2	10.4	44.1	12.4	19.7	5.5	31.6	8.4	78.9
Bloom to maturity	27.7	12.3	42.7	5.7	28.5	7.2	11.0	11.6	27.5	9.2	74.9
Means	34.7	14.2	40.5	7.7	45.0	11.9	28.9	5.1	37.3	9.7	79.0
Production											
Survival	19.6	4.4	15.5	5.6	12.5	2.2	13.2	1.2	15.2	3.3	82.0
Panicles per plant	17.5	6.8	23.6	8.3	21.3	7.3	16.6	7.0	19.8	7.4	72.9
Dry weight	26.4	2.7	27.4	3.4	25.3	3.3	18.9	7.5	24.5	4.2	85.3
Crown area	26.4	2.7	36.5	7.3	11.2	2.7	1.8	5.4	18.9	4.5	80.7
Means	22.5	4.1	25.8	6.2	17.6	3.9	12.6	5.3	19.6	5.4	79.6
Morphology											
Leaf length to width	32.2	7.4	3.9	0.0	15.2	5.4	16.8	5.2	17.0	4.5	79.1
Leaf length × width	57.7	7.0	52.1	12.1	43.0	4.4	25.6	10.9	44.6	8.6	83.8
Plant habit	17.5	19.5	16.1	7.4	26.1	14.9	23.3	19.7	20.7	15.4	57.4
Culm length	30.4	14.8	39.4	12.9	16.3	16.7	16.1	15.7	25.5	15.0	63.0
Panicle length	45.7	13.6	41.4	14.5	25.2	12.8	44.3	12.6	39.2	13.4	74.5
Means	36.7	12.4	30.6	9.4	25.2	10.8	25.2	12.8	29.4	11.4	71.6

### Cultivar comparisons

In most cases, the cultivars as a group did not differ from wild populations (data not shown). Survival, however did differ between groups both in 2009 and 2010 (*P *<* *0.01), averaging 81% for cultivars and 71% for the wild collections. Leaf length × width also differed between groups and was 18% higher for cultivars both years. Dry weight differed between cultivars (26.9 g per plant) and wild populations (20.0 g per plant) only at CF in 2009. Within cultivars, however, there was a range of dry weight with McIntyre, Wallow, Klamath, and Paulina producing more than Reliable and Duffy Creek in 2009 and more than Mountain Home and Reliable in 2010 (*P *<* *0.01); other cultivars were intermediate.

### Correlations among traits and with climate

Pearson linear correlations of traits across garden sites and years were usually moderate to strong indicating considerable correspondence among environments. Among the four site–year combinations, there were six possible correlations; thus, for 15 traits, there were 90 correlations possible. All 90 correlations had positive slopes and 85 of the 90 were highly significant (*P *<* *0.01). The average correlation between traits was *r *=* *0.57 (*P *<* *0.0001, *n* = 126–129), with correlations associated with morphology somewhat higher (*r *=* *0.65) than phenology (*r *=* *0.54) and production (*r *=* *0.53). Although the prevalence of garden site × population interactions indicated plasticity (Table1), the uniformly positive correlations showed these were of magnitude rather than direction. The frequency of significant correlations and their moderate to strong coefficients indicated there was considerable correspondence among traits expressed in different garden sites and years. As a result, data were averaged over years and garden sites, similar to St. Clair et al. (2013), reducing the traits to 15 from a potential of 60. Although some detail was lost through the averaging, it minimized duplication and allowed for a higher probability of finding a few canonical variates that explained a high fraction of the total variance.

Using the wild populations, canonical correlation of the 15 traits resulted in three highly significant (*P *<* *0.01) variates that together explained nearly two-thirds of the variation (Table3). After the third canonical variate, none explained more than 8.2% of the variation or was significant at *P *<* *0.01.

**Table 3:** Summary of canonical correlation between 15 plant traits and 21 climatic variables for Sandberg bluegrass growing at Central Ferry, WA and Powell Butte, OR in 2009 and 2010

Canonical variable	Eigenvalue	Proportion	Cumulative proportion	*F*-Value	*P*-value	Canonical correlation
1	2.58	0.341	0.341	1.95	<0.0001	0.849
2	1.23	0.164	0.505	1.58	<0.0001	0.744
3	1.05	0.139	0.644	1.39	0.0003	0.715
4	0.62	0.082	0.721	1.19	0.0451	0.619
5	0.42	0.056	0.782	1.08	0.249	0.546
6	0.38	0.050	0.832	1.01	0.458	0.523

Pearson linear correlation often linked plant trait variation among source populations with climatic variables (Table4). Significant (*P *<* *0.05) or highly significant (*P *<* *0.01) correlations were observed for continentality (TD) and the annual heat-moisture index (AHM) in 11 of the 15 plant traits (Table4). Correlations for MWMT (mean warmest monthly temperature), TD, mean annual precipitation (MAP), and AHM tended to be stronger and more frequent for phenology and morphology traits than for production traits (Table4). Among canonical variates, the most frequent and strongest correlations with climate were for variate 1 (Table4).

**Table 4:** Pearson correlation coefficients between plant traits on representative climatic and geographic factors for Sandberg bluegrass grown at Central Ferry, WA and Powell Butte, OR

Trait	MAT†	MWMT	MCMT	TD	MAP	MSP	AHM	bFFP	PAS	EXT	Lon.	Lat.	Elev.
Phenology													
Heading	−0.15	−0.27**	0.08	−0.41**	0.42**	0.26**	−0.43**	0.03	0.21*	−0.30**	−0.25**	−0.20*	0.27**
Blooming	0.08	0.00	0.21*	−0.25**	0.26**	0.08	−0.27**	−0.16	−0.02	−0.03	−0.20*	−0.15	0.05
Maturity	−0.11	−0.27**	0.09	−0.42**	0.48**	0.32**	−0.49**	−0.06	0.24**	−0.31**	−0.28**	−0.02	0.11
Head to bloom	0.40**	0.50**	0.21*	0.31**	−0.36**	−0.39**	0.33**	−0.30**	−0.45**	0.49**	0.14	0.04	−0.35**
Head to bloom	0.14	0.19*	−0.03	0.25**	−0.24**	−0.15	0.24**	−0.09	−0.14	0.20*	0.14	0.26**	−0.31**
Bloom to mat.	−0.19*	−0.21*	−0.20*	0.01	0.00	0.12	−0.01	0.18*	0.20*	−0.19*	0.06	0.18*	0.01
Production													
Survival	0.09	0.26**	−0.06	0.37**	−0.43**	−0.34**	0.42**	0.09	−0.30**	0.34**	0.36**	−0.19*	0.05
Panicles	0.11	0.09	0.15	−0.07	−0.08	−0.14	0.01	−0.06	−0.21**	0.08	−0.03	−0.12	−0.05
Dry weight	0.01	−0.06	0.08	−0.16	0.12	0.10	−0.25**	−0.10	−0.02	−0.05	−0.02	0.07	−0.09
Crown area	−0.08	−0.15	0.02	−0.20*	0.23**	0.23**	−0.31**	−0.07	0.15	−0.18*	−0.03	0.11	−0.04
Morphology													
Leaf lnth to width	0.39**	0.43**	0.20*	0.25**	−0.16	−0.31**	0.31**	−0.37**	−0.34**	0.40**	0.02	0.13	−0.42**
Leaf lnth × width	−0.11	−0.20*	0.00	−0.23**	0.38**	0.26**	−0.43**	−0.14	0.25**	−0.17	−0.12	0.10	−0.02
Habit	0.07	0.03	0.19*	−0.20*	0.00	−0.17	−0.07	0.06	−0.19*	−0.03	−0.25**	−0.39**	0.23**
Culm length	0.00	−0.06	0.09	−0.18*	0.10	0.07	−0.23**	−0.03	−0.07	−0.02	−0.08	−0.08	0.05
Panicle length	0.09	0.06	0.12	−0.09	0.08	−0.01	−0.12	−0.18*	−0.09	0.06	−0.04	−0.03	−0.08
CanCorr1	−0.35**	−0.53**	−0.02	−0.58**	0.60**	0.47**	−0.65**	0.18*	0.47**	−0.56**	−0.33**	−0.14	0.38**
CanCorr2	−0.14	−0.18	−0.15	−0.02	0.19*	0.37**	−0.17	−0.08	0.32**	−0.17	0.08	0.41**	−0.17
CanCorr3	0.31**	0.20*	0.27**	−0.09	0.23**	−0.03	−0.15	−0.44**	−0.06	0.14	−0.25**	0.24**	−0.42**

*** Significant at *P *<* *0.05 (*r* > ± 0.18) and *P *<* *0.01 (*r* > ± 0.23), respectively, *n* = 130.

† Annual climatic variables as given by Wang et al. (2012) as MAT (mean annual temperature), MWMT (mean warmest month temperature), MCMT (mean coldest month temperature), TD (continentality MWMT- MCMT,), MAP (mean annual precip.), MSP (mean summer precip. May–September), AHM (annual heat moisture index [MAT + 10°C]/[MAP mm/1000]), bFFP (day of year frost free period begins), PAS (precip. as snow), EXT (extreme max. temperature over 30 years), Lon. (longitude), Lat. (latitude), Elev. (elevation).

Heading, blooming, and maturity dates were generally later with low TD, high MAP, and at source populations with high AHM. For survival, there were positive correlations with TD and AHM and the negative correlations with MAP and mean summer precipitation (MSP), indicating more survival in gardens of plants from warmer, dryer source populations with large seasonal changes in temperature than those from wetter, more moderate temperatures (Table4). For morphology, leaf characteristics exhibited associations with climate consistent with natural selection. High leaf length to width ratio, indicating relatively narrow leaves and promoting sensible heat loss during plant development, was associated with higher MAT and MWMT. For precipitation, the leaf to width ratio was higher with less MSP, higher AHM, an earlier end to frost in the spring (lower bFFP), and less precipitation as snow (PAS) (Table4).

### Regression modeling and mapping

Regression models of significant canonical variates (Table3) for phenology (heading), production (dry weight), and morphology (leaf to width ratio and leaf × width) traits with climate were all highly significant (*P *<* *0.0001) with *R*^2^ values between 0.27 and 0.71 (Table5). The high *R*^2^ for regression of canonical variate 1 (0.71) was associated with its relatively high and frequent correlation with climatic variables (Table4) showing the association between genetic variation and climate.

**Table 5:** Coefficients of determination (*R*^2^) and equations for regression of plant traits with climate variables at source populations of Sandberg bluegrass

Trait*	*R*^2^	Regression equations†
Can 1	0.71	−55.2451 + 2.85191(MAT) + 0.29718(MCMT) + 0.0011(MAP)−0.00555(MSP)−0.00592(SHM)−0.00592(DD > 5) + 0.01007(DD < 18) + 0.05548(NFFD)−0.07434(eFFP) + 0.04266(FFP)−0.12612(EMT) + 0.00836(Eref)
Can 2	0.51	156.3392−8.19334(MAT)−0.30254 (MCMT)−0.01115(SHM)−0.00637(DD < 0)−0.02325(DD < 18) + 0.0244 (DD > 18) + 0.00491(PAS)−0.00953(Eref) + 0.00761(CMD)
Can 3	0.48	−91.1491 −0.2078(TD)−0.02946(AHM) + 0.00553(SHM)−0.01617(DD < 0) + 0.02297(DD > 5) + 0.01762(DD < 18)−0.02338(DD > 18) + 0.05582(NFFD)−0.06425(eFFP)
Heading date	0.46	−0.26944 + 3.55225(MCMT) + 0.7232(TD) + 0.01197(MAP)−0.03614(SHM)−0.02346(DD < 0) + 0.0342(DD < 18) + 0.31645(NFFD)−0.56347(eFFP) + 0.27197(FFP)−0.02621(PAS)−0.96566(EMT) + 0.02973(CMD)
Biomass	0.27	−71.7559−0.0706 (MSP)−0.1184 (SHM)−0.0553(DD < 0) + 0.6545(NFFD)−0.2759(FFP)−2.8993(EMT) + 1.6127 (EXT)−0.0850(Eref)
Leaf length to width	0.35	94.8648 + 1.5622(TD) + 0.01404(MAP) + 0.02579(SHM)−0.00753(DD < 18)−0.1898(eFFP)−0.04176(PAS)−0.03407(Eref)
Leaf length × width	0.36	3.83528 + 0.00112(MAP)−0.00704(MSP)−0.00581(SHM)−0.00181(DD < 0)−0.01971(bFFP)−0.07135(EMT) + 0.07433(EXT)−0.00294 Eref

* Can 1, 2, and 3 are the first three canonical correlation variates based on plant traits measured in common gardens.

† All regression models were significant at *P* < 0.0001. Annual climatic variables as given by Wang et al. (2012) were MAT (mean annual temperature), MWMT (mean warmest month temperature), MCMT (mean coldest month temperature), TD (continentality MWMT- MCMT,), MAP (mean annual precip.), MSP (mean summer precip. May–September), AHM (annual heat moisture index [MAT+10°C]/[MAP/1000 mm]), SHM (summer heat moisture index MWMT/[MSP/1000 mm]), DD < 0 (chilling degree days, degree days below 0°C), DD > 5 (growing degree days, degree days above 5°C), DD < 18 (heating degree days, degree days below 18°C), DD > 18 (cooling degree days, degree days above 18°C), NFFD (frost free days), FFP (frost free period), bFFP (day of year FFP begins), eFFP (day of year FFP ends), PAS (precip. as snow), EMT (30-year extreme min. temp.), EXT (30-year extreme max. temp.), Eref (Hargreaves reference evaporation), CMD (Hargreaves climatic moisture deficit).

Maps of plant traits revealed populations from warmer and drier areas, such as the Columbia Plateau and much of the Central Basin and Range, usually had less dry weight, earlier heading, and smaller, more narrow leaves (Fig.1). Most often, populations with higher dry weight had later heading (*r *=* *0.43, *P *<* *0.01) and higher leaf area (*r *=* *0.69, *P *<* *0.01). Plants with the highest leaf to width ratios (relatively narrower leaves) were from dry areas with typically lower dry weights, such as the central Columbia Plateau, the lower Snake River Plain, and the eastern Central Basin and Range. But plants with higher leaf to width ratios were also from wetter areas such as the Klamath Mountains that supported higher plant dry weights (Fig.1). As a result, dry weight and leaf to width ratios did not correlate (*r *=* *0.13).

**Figure 2:**
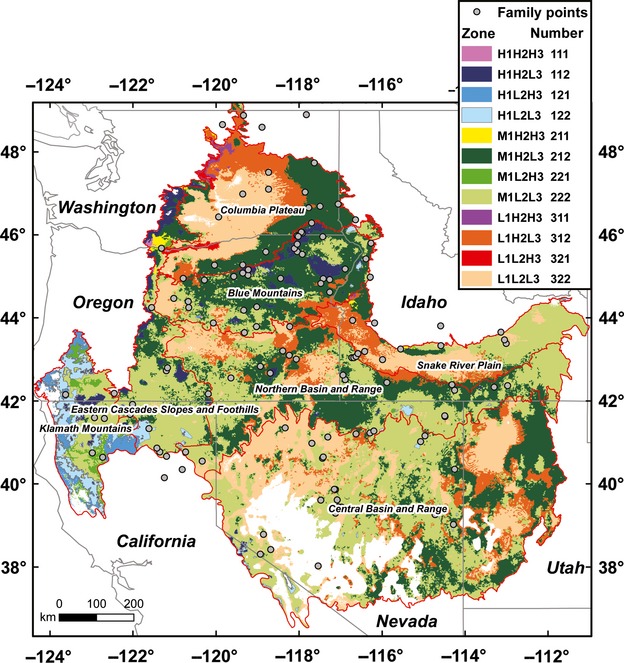
Twelve proposed seed zones for Sandberg bluegrass over ecoregions based on regression models between plant trait canonical variates 1, 2, and 3 and climate variables over intermountain west level III Omernik ecoregions and the Klamath Mountain ecoregion. Ecoregion boundaries are shown as red lines. Canonical variate 1 values were represented by high (H_1_), middle (M_1_), and low (L_1_) sections, and variates 2 and 3 by high (H_2_ and H_3_) and low (L_2_ and L_3_) sections. The circles show collection source populations. Model predictions outside the data range for traits were not mapped, and were shown in white.

The overlay of the three significant canonical variates (Table3), achieved by dividing variate 1 values into sections high (H_1_), middle (M_1_), and low (L_1_), and variates 2 and 3 into high (H_2_ and H_3_) and low (L_2_ and L_3_) sections, resulted 12 seed zones (Fig.2). Canonical variate 1 was weighted more heavily (divided into three sections instead of two) owing to its higher *R*^2^ in the canonical correlation (Table3). A total of 700 981 km^2^ was mapped. The smallest, zone 111 (pink), covered only 298 km^2^ and was almost indiscernible at our mapping scale. Four zones represented nearly 92% of the mapped area: 212 (dark green) with 26.6%, 222 (light green) with 35.5%, 312 (brown–orange), with 10.2%, and 322 (tan) with 19.3% (Fig.2).

**Figure 3:**
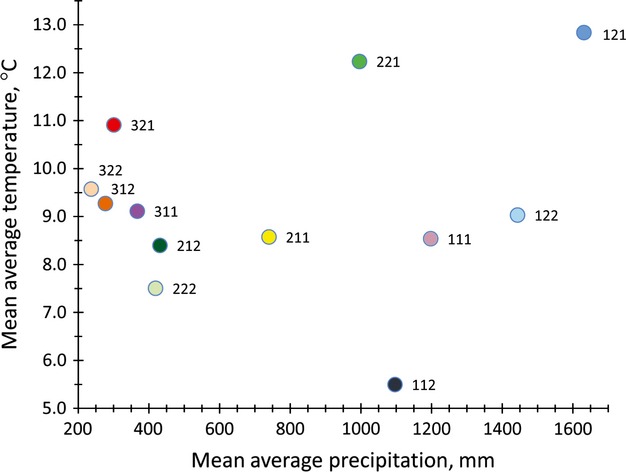
Relationship between average temperature and precipitation for seed zones developed from plant traits and climate variables for Sandberg bluegrass.

Seed zones could be divided on the basis of precipitation as relatively moist (>995 mm), mesic (740 mm), semi-arid (367–431), and arid (237–301) (Fig.3). The Klamath Mountain ecoregion (Omernik 1987) was represented by five seed zones (Fig.2). Positioned between Coast Range and Cascade ecoregions, the Klamath Mountain ecoregion it is not technically part of the intermountain region. The high precipitation zones 121 (mid-blue) and 122 (light blue) predominantly occurred in the Klamath Mountain ecoregion, as did the semi-arid 221 (mid-green) (Fig.2). Thus, the Klamath Mountains, with pacific maritime influence and varied topography, were particularly diverse climatically as well as genetically for Sandberg bluegrass. Transition from arid zones 312 (brown–orange) and 322 (tan) to semi-arid zones 212 (dark green) and 222 (light green) was associated with higher dry weight, later heading, lower leaf to width ratios, and higher leaf area (Figs1 and 2).

Differences in precipitation and temperature within the semi-arid zones 212 and 222 and the arid zones 312 and 322 were relatively small (Fig.3), indicating that plant traits can evolve in response to fine-scale differences in climate. For example, zone 212 usually represented populations with higher dry weight, later heading, and higher leaf area than zone 222 (Figs1 and 2), with a similar pattern observed between zone 312 and 322.

## Discussion

Genetic variation for all plants traits measured was always observed in common gardens, and climatic factors at source populations were frequently linked to genetic traits. The pattern of genetic variation with differing climates was logical in terms of expected adaptations to high temperature and drought stress environments common in the intermountain west (Levitt 1980; Westoby et al. 2002; Warren et al. 2006; Maricle et al. 2007; St. Clair et al. 2013). In higher stress environments, earlier heading promotes escape from higher temperatures and summer droughts and narrower leaves promote a smaller boundary layer and higher sensible heat transfer, promoting improved temperature regulation. Less leaf area, if occurring on a whole plants basis, reduces the potential for transpirational water loss. On the other hand, source populations from relatively cooler, wetter environments had generally later phenology, somewhat higher biomass, and relatively wider leaves with more leaf area, attributes that would likely more fully utilize lower stress environments (Table4 and Fig.1). However, Sandberg bluegrass from very warm and moist environments, such as zone 121 (mid-blue) in the Klamath Mountain ecoregion, had relatively narrow leaves coupled with higher leaf area, an apparent adaptation to higher temperatures independent of frequent and severe droughts (Figs1 and 3). Still, predicated average dry weights in the high moisture areas never exceeded those of the semi-arid zone 212 (dark green), a climate more typical of Sandberg bluegrass habitat (Figs1 and 2).

Genetic variation in wild populations of grasses and forbs has been reported for species from the intermountain west (Johnson et al. 2010, 2012, 2013; St. Clair et al. 2013) and is generally observed in common garden research in other regions (St. Clair et al. 2005, Leimu and Fischer 2008; Horning et al. 2010). Differences among traits between source populations at different garden environments showed the expected presence of plasticity (Johnson et al. 2013, St. Clair et al. 2013). Still, within the common gardens, the strong differences among source populations for all traits indicated the presence of extensive genetic variation.

We expected trait differences between cultivars and wild populations. St. Clair et al. (2013) found five commercial releases of bluebunch wheatgrass (*Pseudoroegneria spicata* (Pursh) Á. Löve) had higher dry weight and more inflorescences than wild collected material from the intermountain west, the apparent result of agricultural selection. In our experiment, cultivar survival was 10% higher and leaf area was 18% higher than the wild populations as a whole (*P *<* *0.01), suggesting direct or inadvertent selection for those traits. Cultivar survival would be desirable for establishment and might relate to seedling vigor, a trait highly valued in plant material development (Leger and Baughman 2014). Higher leaf area was apparently favored in cultivar selection, an attribute our analysis suggests might result in plant material less suited to dryer environments. For all year–site combinations cultivar dry weight was higher than wild populations only at CF in 2009. Still, individual cultivars varied strongly for dry weight (*P *<* *0.01). We did not see the generally high dry weight and inflorescence number for cultivars that St. Clair et al. (2013) observed for bluebunch wheatgrass. This suggested agricultural selection for broad adaptation in the outcrossing bluebunch wheatgrass was more prevalent than for apomictic Sandberg bluegrass. Given the different climates represented in this region and the degree of local adaptation we found in wild collections, using seeds from the appropriate seed zone for restoration is likely a more optimal approach than widespread use of a few cultivars.

In this study, 77% of total source population and family variance was associated with source populations, similar to the 81% observed by Erickson et al. (2004) for the strongly self-pollinating blue wildrye (*Elymus glaucus* Buckley). Larson et al. (2001) found a high amount of AFLP marker variation within two geographically distant Sandberg bluegrass populations but little divergence between populations. Their research suggested that a few population sources of Sandberg bluegrass could potentially be used for restoration without compromising overall genetic variability. But variation in neutral markers is not normally an appropriate surrogate for adaptive diversity (McKay and Latta 2002). This was illustrated by our results; phenotypic traits exhibited a generally high among source population variability with much less within population variability. With facultative apomixis, natural selection and adaptation to specific environments is likely promoted over time by sexual reproduction, with adaptive variation maintained within diverse environments (Kellogg 1987; Kelley et al. 2009). The mating system of Sandberg bluegrass combined with the extensive genetic variation for different plant traits suggests response to environmental selective forces leading to evolution and differences in adaptation among Sandberg bluegrass populations.

By averaging traits over garden environments and reducing the number of plant trait variables for canonical correlation from 60 to 15, some information regarding phenotypic plasticity was lost that could be important for plant performance in specific microclimates. However, strong positive correlations among traits and garden site–year environments showed that plasticity was of magnitude rather than direction and that genetic variation was consistently expressed. Moreover, nearly two-thirds of variation between the average plant trait and climate was explained by the three highly significant canonical variates (Table3). Both the canonical correlation analyses (Table3) and linear correlation analysis (Table4) of averaged traits established a relationship between genetic diversity and climatic variability among source populations. This is consistent with other genecology studies of bunchgrasses in the intermountain west (Johnson et al. 2010, 2012; St. Clair et al. 2013). In this study, we focused on genetic variation, but more detailed investigations of the observed plastic variation, potentially also under genetic control (Bradshaw 1965), would advance the understanding of its overall importance to adaptation (Scheiner and Goodnight 1984).

Regression of canonical traits with climate resulted in models with *R*^2^ values ranging from 0.48 to 0.71 and was comparable or stronger than reported for other genecology studies of perennial grasses in the intermountain west. For bluebunch wheatgrass, St. Clair et al. (2013) found *R*^2^ values of principal components (PCs) scores regressed on climate of 0.28 and 0.51. Erickson et al. (2004) reported regression *R*^2^ values with PCs of 0.30 and 0.51 for blue wildrye (*Elymus glaucus* Buckley) models, and Johnson et al. (2010) reported values of 0.40 and 0.46 for mountain brome. For Indian ricegrass (*Achnatherum hymenoides *(Roem. & Schult.) Barkworth), regression models with canonical variates and climate ranged from 0.16 to 0.43 (Johnson et al. 2012). Among conifers Douglas fir is considered closely adapted to differing environments and regression models between canonical variates and climate resulted in *R*^2^ values of 0.50 and 0.68 (St. Clair et al. 2005). Horning et al. (2010) developed a regression model for oceanspray (*Holodiscus discolor* (Pursh) Maxim.) from the first PC and climate with a *R*^2^ of 0.86. Thus, our regression models for Sandberg bluegrass were comparatively strong and an appropriate basis for seed zone development.

In this experiment, we used transplants to promote establishment for whole-plant evaluation. In doing so, we were unable to examine establishment traits such as germination and emergence. Given that genetic variation in whole-plant traits was linked to climate perhaps differences in seed recruitment would also be linked. Espeland and Hammond (2013) found Sandberg bluegrass performance at early growth stages were positively correlated with later stages. However, as transplant success far exceeds that of direct seeding, transplanting should be considered for Sandberg bluegrass and other restoration species. Maternal effects could also have affected population traits in different environments. Espeland and Hammond (2013) demonstrated adaptive maternal effects in Sandberg bluegrass for some traits and production environments, but not consistently and not in later growth stages. In their study, seed size did not affect any life-history stage past germination, having no discernible influence on emergence or biomass accumulation.

Depending on the needs of land managers, there are many cases when smaller scale restoration maybe desired for specific needs. More mapping detail could be achieved by dividing the range of canonical variates into more sections. This rapidly increases the number of zones and also the complexity of maps and the cost of additional collection and seed increase. In the current seed zone map as in similar studies (St. Clair et al. 2013), we sought to balance seed zone size with the practical needs of *in situ* application on relatively large-scale restoration projects. As the scope for future selective adaption and evolution depends on the availability of genetic variation (Sgrò et al. 2011), we also recommend numerous wild populations be collected and used for restoration within seed zones.

Genecological studies provide information regarding the ability of plant populations to evolutionarily respond to climate variation. Here, we show that Sandberg bluegrass expresses distinct multivariate phenotypes that likely evolved across environmental gradients that were, at times, quite small. Our research supports preferential use of restoration seeds that are appropriately sourced. Although seeds that are juxtaposed geographically are often used when local, specific adaptation is a concern, we show that adaptation on a climatic scale may be used to inform seed source choices, especially in larger scale restoration. The seed zones developed will provide guidance for the choice of genetic resources for restoration, and compared to using a limited number of cultivars, promote *in situ* genetic variation across the landscape.
